# Disseminated *Lomentospora prolificans* infection in a neutropenic patient with acute monocytic leukemia: a clinical and diagnostic challenge

**DOI:** 10.1128/asmcr.00093-25

**Published:** 2025-09-25

**Authors:** Sierra Amaya, Giancarlo Giovannini-Sanguineti, Corina Lopez, Carolyn D. Alonso, Stefan Riedel

**Affiliations:** 1 Department of Pathology, Beth Israel Deaconess Medical Center 376938 , Boston, Massachusetts, USA; 2 Department of Medicine, Division of Infectious Diseases, Beth Israel Deaconess Medical Center 1859 , Boston, Massachusetts, USA; 3 Harvard Medical School 1811 , Boston, Massachusetts, USA; Vanderbilt University Medical Center, Nashville, Tennessee, USA

**Keywords:** *Lomentospora prolificans*, disseminated fungal infection, neutropenia, acute monocytic leukemia, antifungal resistance, immunosuppression, invasive fungal infection (IFI)

## Abstract

**Background:**

*Lomentospora prolificans* is an opportunistic fungal pathogen associated with significant morbidity and mortality in immunocompromised individuals. Its intrinsic resistance to most antifungal agents poses challenges in clinical management, particularly in patients with hematological malignancies.

**Case Summary:**

Herein, we report the case of a 60-year-old male with recently diagnosed acute monocytic leukemia who developed a disseminated *L. prolificans* infection during induction chemotherapy. Despite standard prophylactic antimicrobials, the patient experienced febrile neutropenia, progressive pulmonary findings, and neurological complications, culminating in disseminated fungal infection. Blood cultures confirmed the diagnosis, and antifungal therapy was promptly expanded. However, the infection progressed rapidly, and the patient died despite aggressive therapeutic interventions.

**Conclusion:**

This case highlights the complexities of diagnosing and treating *L. prolificans* infections in profoundly immunosuppressed patients. It underscores the importance of heightened clinical suspicion, early recognition, aggressive management, and the need for continued development of novel antifungal agents and diagnostic techniques.

## INTRODUCTION

*Lomentospora prolificans*, formerly known as *Scedosporium prolificans*, is a globally emerging opportunistic pathogen that poses significant challenges in clinical management due to its intrinsic resistance to most antifungal agents and its association with high mortality rates. This filamentous fungus has been identified in sources such as soil and sewage and affects immunocompromised individuals, particularly those with hematological malignancies and solid organ transplants (1–4).

*L. prolificans* infections manifest in various forms, including fungemia, pneumonia, skin and soft tissue infections, and disseminated disease. The pathogen can cause breakthrough or persistent infections despite antifungal prophylaxis, as seen in heart transplant recipients and hematopoietic stem cell transplant patients (2, 5). The clinical presentation often involves rapid progression and severe complications, such as central nervous system involvement and endocarditis, which contribute to the poor prognosis (3).

Diagnosis of *L. prolificans* infections is challenging due to the histological overlap in morphology with other hyaline molds (6, 7). Accurate identification typically requires a combination of culture, histopathology, and molecular techniques. Treatment options are limited, with combination antifungal therapy being the current standard approach. Agents such as voriconazole and terbinafine have shown some efficacy, but the overall success rates remain low (3). Novel antifungal agents such as olorofim and fosmanogepix are under investigation and hold promise for future therapeutic strategies (1).

We report a case of disseminated *L. prolificans* infection in a patient with acute monocytic leukemia. This case highlights the complexities of diagnosing and treating invasive fungal infections (IFIs) in the setting of profound immunosuppression and underscores the need for continued advancements in antifungal therapeutics and diagnostic strategies.

## CASE PRESENTATION

A 60-year-old male with type 2 diabetes mellitus, hypertension, and hyperlipidemia was admitted to the hospital for management of newly diagnosed acute monocytic leukemia. He initially presented to an outside hospital for evaluation of progressive bilateral shoulder, head, and neck pain and was found to have leukocytosis (35,140 WBC/µL, ref. range 4-10k cells/µL) with 44% of blasts on peripheral blood. The patient was started on induction chemotherapy with cytarabine/daunorubicin (day 0) after the diagnosis was confirmed by bone marrow biopsy. Per our institutional protocol, he was started on prophylactic antimicrobial treatment with levofloxacin (500 mg PO q24h), acyclovir (400 mg PO q12h), and posaconazole (300 mg PO q24h). He became severely neutropenic (ANC <500/µL, ref. range 1.6 k–6.1k cells/µL) on day 3 of induction chemotherapy. His hospital course was complicated by profound hypocalcemia, severe diarrhea, and orthostatic hypotension. On day 10, a computed tomography (CT) scan of the chest showed “tree-in-bud” nodularity in the left lower lobe, in the right upper and middle lobes, which had been incidentally noted on a CT scan of the abdomen and pelvis on that same day. On day 12, the patient developed febrile neutropenia, prompting an evaluation for an infectious etiology. He endorsed a non-productive cough, headaches, and persistent intermittent abdominal pain. The initial infectious disease evaluation included a chest X-ray and laboratory testing, including an extended viral respiratory viral panel (cobas ePlex RP-2 panel, Roche Diagnostics), urinalysis, urine culture, a serum 1,3-beta-D-glucan (BDG) test, and a serum *Aspergillus* galactomannan (GM) Ag test, as well as aerobic and anaerobic blood cultures (BC). All of these tests were negative. At that point, antimicrobial therapy with levofloxacin was transitioned to cefepime (2 g IV q8h). On day 13, the patient was started on metronidazole given persistent fevers and evolving abdominal pain. A CT angiogram of the abdomen and pelvis (on day 16) showed soft tissue stranding of the mesentery with prominent lymph nodes as well as worsening left lower lung lobe consolidative opacity. Given persistent fevers, the Infectious Diseases team was consulted, and cefepime and metronidazole were changed to piperacillin-tazobactam (4.5 g IV q8h). On day 17, the patient developed altered mentation with a new episode of hallucinations, new dyspnea, and chest tightness. Intravenous Vancomycin (1.25 g IV q8H) was started for concern of worsening pneumonia and to include coverage for additional Gram-positive bacterial organisms. At that time, a magnetic resonance imaging (MRI) scan of the brain with and without contrast was within normal limits. On day 19, the patient experienced rigors with fever, elevated lactate (5.2 mmol/L, ref. range 0.5–2.0 mmol/L), and new oxygen requirement (1.5L NC). Piperacillin-tazobactam was broadened to meropenem while vancomycin was continued. On day 20, a repeat chest CT scan showed new consolidative opacities in all lobes. The absolute neutrophil count was noted to be rising above 500 cells/µL. *Legionella pneumophila* and *Streptococcus pneumoniae* urine antigen tests were negative. On day 22, he had an acute episode of confusion with right-sided ptosis. A head CT scan showed new curvilinear hyperdensity along the postcentral sulcus that was concerning for small subarachnoid hemorrhage as well as new hypodensities in the bilateral cerebellar hemispheres, right caudate nucleus, and white matter of the right parietal lobe that could represent infarcts.

**Fig 1:**
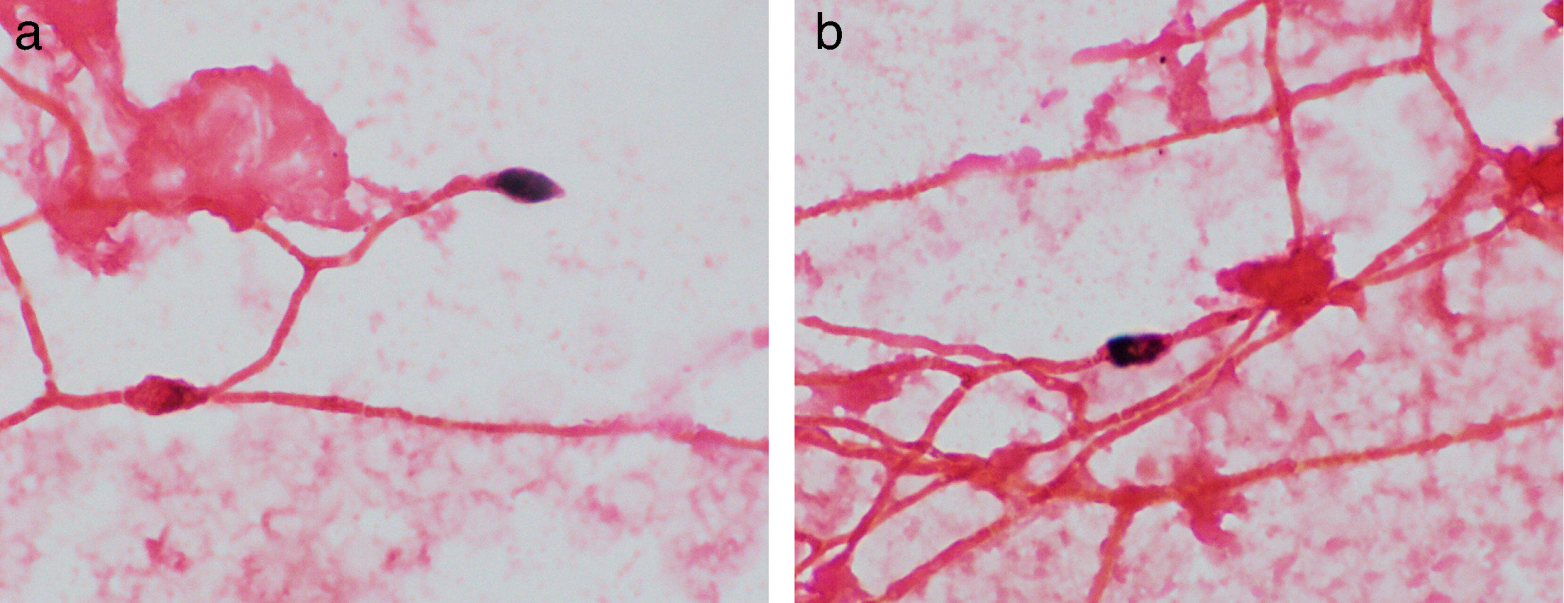
(**a and b**) Gram stain from the original blood culture bottle (magnification: ×1,000). The Gram stain shows branching septate hyphae with occasional visualization of a single conidium. The latter should not be confused with yeast cells, as they are larger and show no budding.

**Fig 2:**
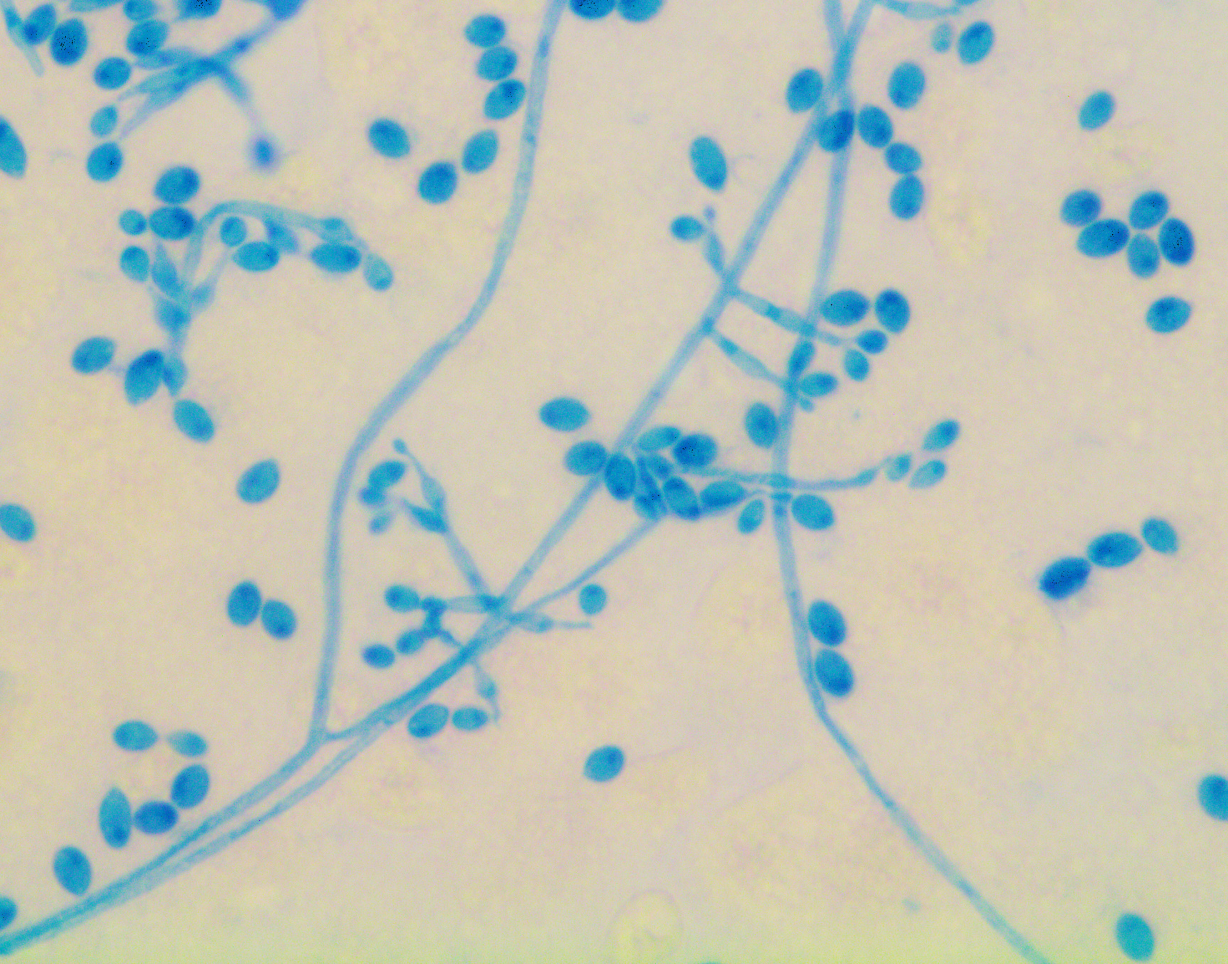
Lactophenol Cotton Blue (LPCB) stain of *L. prolificans*. LPCB stain shows delicate, septate hyphae. The hyphae exhibit irregular branching and produce conidiogenous cells with a swollen base and an elongated neck that bear single or clustered, oval to ellipsoid conidia. The one-celled conidia are typically smooth-walled, ovoid, and have a slightly narrowed, truncated base. These features help distinguish *L. prolificans* from other filamentous fungi, making LPCB a useful tool in its identification.

**Fig 3:**
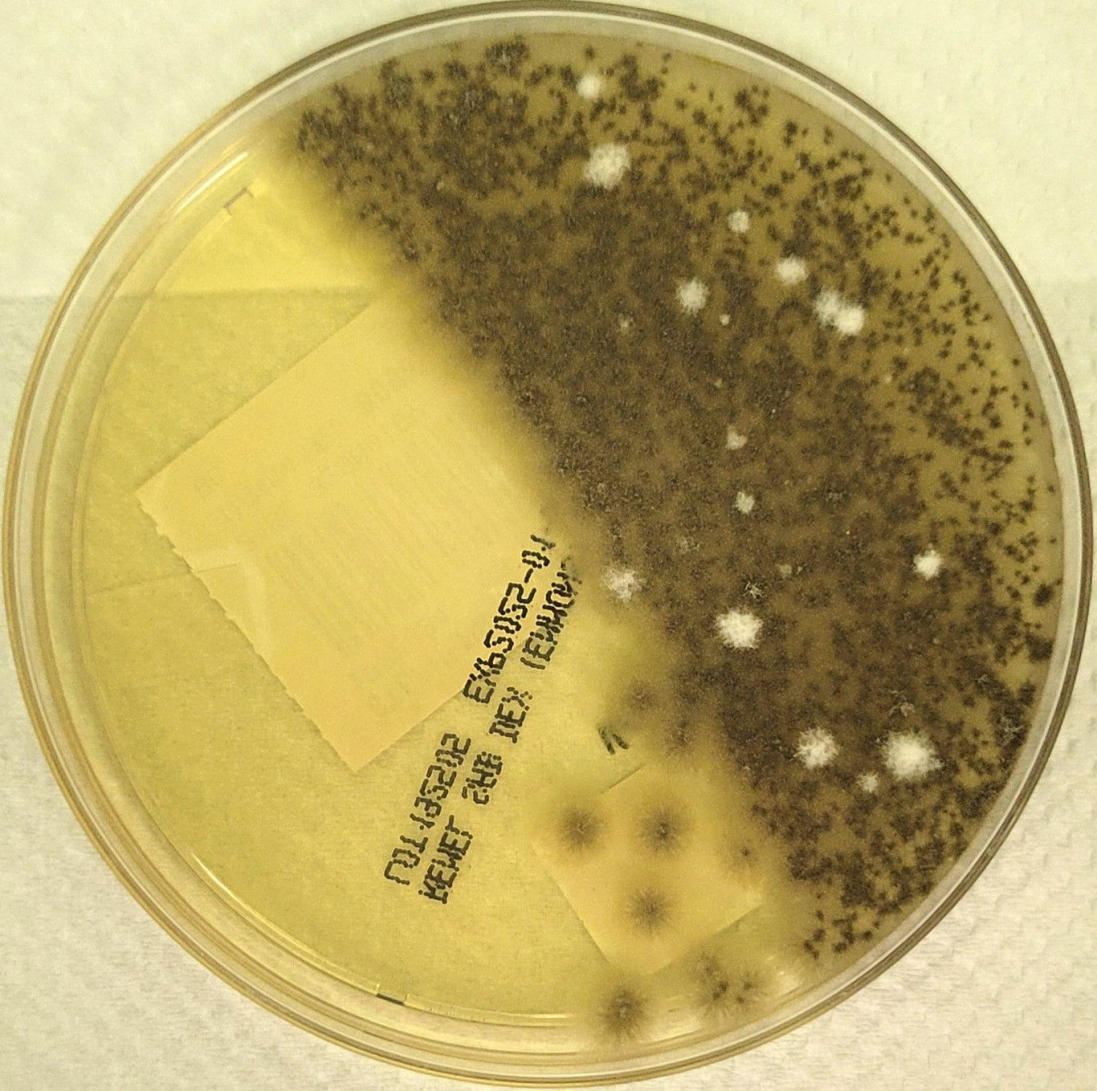
*L. prolificans* growth on Sabouraud Dextrose Agar showing characteristic colonies of *L. prolificans*; young colonies are cottony and light gray to black, whereas mature colonies become dark gray to black and may develop white mycelial tufts, as shown in the image here. The reverse side is gray to black.

On day 24, the BC collected on day 20 was positive, and fungal elements were seen on Gram stain from the aerobic bottle (Fig. 1). The Gram stain demonstrated septate hyphal elements with possible conidia being present, therefore raising a concern for a *Scedosporium/Lomentospora spp*. or a *Fusarium spp*. The same day, posaconazole was discontinued and the patient was started on liposomal amphotericin B (5 mg/kg) and voriconazole (6 mg/kg load, followed by 4 mg/kg). The following morning, repeat 1,3-Beta-D glucan was elevated (>500 pg/mL, ref. range <60 pg/mL). The serum GM Ag test remained negative (ref. range <0.5). The transthoracic echocardiogram was unremarkable. The patient continued to experience episodic altered mentation and fevers. Brain MRI with and without contrast showed multiple diffusion restrictions with FLAIR hyperintensity in the bilateral cerebral and cerebellar hemispheres, consistent with multiple acute infarcts as well as hemorrhagic transformation in the right occipital lobe infarct. A lumbar puncture was performed which showed CSF pleocytosis with 223 nucleated cells (ref. range <5cells/µL), 76% of which were neutrophils, 348 red blood cells, glucose of 63 mg/dL, and an elevated protein (66 mg/dL, ref. range 15–45 mg/dL). The CSF Gram stain was negative for microorganisms, and a routine CSF culture was performed. The CSF eventually was negative for bacterial and/or fungal growth. The BC showed growth of a mold on the sheep blood agar plate; subculture on Sabouraud Dextrose agar was set up, and the mold was identified as *Lomentospora prolificans* on day 26, based on morphological characteristics at 72 h of growth (Fig. 2 and 3). Antifungal susceptibility testing was performed at our institution’s reference laboratory, and results are shown in Table 1. The liposomal amphotericin B dose was increased to 7 mg/kg. Terbinafine (500 mg PO q12h) and micafungin (150 mg IV q24h) were added to the treatment regimen. However, on the next day, the patient had worsening of his mental status, profound somnolence, and was difficult to arouse, with a report of repeated aspiration events. A CT head scan showed new acute infarcts in the frontotemporal lobe. Given the need for escalation of care, a goals-of-care conversation was held with the family. At that point, it was decided to continue with comfort measures only; the patient expired 2 days later, on day 29 of his hospitalization.

**TABLE 1:** *L. prolificans* antifungal susceptibility testing results

Antifungal agent	Result (mcg/mL)^*a*^
Amphotericin B	>16
5-Fluorocytosine	>64
Micafungin	>8
Posaconazole	>16
Voriconazole	>16
Isavuconazole	>16
Terbinafine	>2
Manogepix	0.015
Olorofim	0.06

^*a*^ There are no established interpretive criteria for the antifungal agents tested here.

## DISCUSSION

The incidence of IFIs in patients with acute leukemia is significantly high, with neutropenia serving as a primary risk factor with *Aspergillus* species being the most frequently implicated pathogens (8–10). In this case presented here, the patient’s prolonged neutropenic state, coupled with the finding of pulmonary nodularity on the CT scan, strongly suggested an IFI, despite negative initial diagnostic testing for 1,3-beta-D-glucan and GM blood tests.

1,3-beta-D-glucan and galactomannan are cell wall components of different fungal species that can be detected in serum and are used to aid in the diagnosis of IFIs. In invasive infections with *Scedosporium* spp. and *Lomentospora* spp. BDG has been found to have higher sensitivity than GM (81.5% vs 27%) and can precede the diagnosis by conventional methods in up to 94% of cases (11). However, these tests lack specificity and should not be used in isolation. In our case, the positive BDG and negative GM Ag tests were suggestive of an IFI, but direct visualization and recognition of fungal elements such as hyphae and conidia on blood culture Gram stains were fundamental for rapid identification.

*Lomentospora prolificans* and *Scedosporium apiospermum* are ubiquitous fungi found in environmental sources (e.g., in soil and decaying vegetation), and their clinical manifestations range from colonization of the respiratory tract, invasive localized disease, to disseminated infections (12–14). While fungemia due to these two organisms is infrequently seen in BCs in clinical laboratories, the fungal conidia seen on the BC Gram stain can be initially mistaken for yeast cells. The recognition of truncated conidia along with septate hyphae is an important distinguishing feature from yeast cells forming pseudohyphae. Other than *L. prolificans* as seen in this case, *Scedosporium apiospermum*, *Aspergillus terreus,* and *Fusarium* species are molds that produce conidia directly on hyphae *in vivo*, and therefore can be recognized on blood culture Gram stains. In the case presented here, the visualization of microscopic characteristics of *L. prolificans* on the BC Gram stain allowed for early recognition and consideration of this organism, prompting the clinical team to make appropriate therapeutic changes.

However, managing IFIs in neutropenic patients is challenging, given the limited efficacy of available antifungal agents and the rapid disease progression often seen in this population. According to the National Comprehensive Cancer Network guidelines, clinicians must maintain a high level of suspicion and initiate early antifungal therapy in high-risk patients, such as those undergoing intensive chemotherapy for acute leukemia (12, 15–17). Empirical antifungal therapy is typically recommended in the presence of clinical or radiological findings suggestive of an IFI, even in the absence of confirmatory microbiological evidence. Invasive infection from multidrug-resistant *Lomentospora prolificans* is associated with higher mortality, particularly among immunocompromised hosts. Although voriconazole is considered the antifungal agent of choice, the MICs against voriconazole observed in various studies are high. Resistance to first-line antifungal agents (including liposomal amphotericin B and voriconazole) is common, and thus presents challenges with upfront empiric antifungal therapy selection (18). In some case reports, combination therapy of voriconazole with terbinafine has shown some success, albeit the exact role of such combination therapy has not been fully established (19). In this case, the echinocandin micafungin was added to the empiric antifungal regimen, which included liposomal amphotericin for a potential synergistic effect (20). Newer antifungal therapies such as manogepix and olorofim are promising treatments, though access and clinical outcomes data are currently limited.

In this case, the patient’s persistent febrile neutropenia, combined with evolving pulmonary findings and the onset of new neurological symptoms, necessitated an aggressive diagnostic and therapeutic approach. Early diagnostic imaging, timely initiation of empirical antifungal therapy, and careful monitoring of the clinical response are critical to improving outcomes. The patient’s profound neutropenia and complex clinical presentation underscore the necessity for heightened vigilance for IFIs and the need for prompt, evidence-based interventions (15, 16, 21).
